# The IMPAKT of breast cancer research: fundamental science and applied medicine

**DOI:** 10.4155/fso.15.69

**Published:** 2015-09-11

**Authors:** Heloisa Helena Milioli

**Affiliations:** 1 Priority Research Centre for Bioinformatics, Biomarker Discovery & Information-Based Medicine, Hunter Medical Research Institute, New Lambton Heights NSW, Australia; 2 School of Environmental & Life Sciences, The University of Newcastle, Callaghan NSW, Australia

**Keywords:** breast cancer, clinical applications, conference, ESMO, fundamental research, IMPAKT, patient care, translational science

## Abstract

The IMPAKT 2015 Breast Cancer Conference was designed for researchers and clinicians by the Breast International Group (BIG) and the European Society for Medical Oncology (ESMO). The event was held on 7–9 May in Brussels, Belgium, bringing together approximately 525 participants with a special interest in translational science and state-of-the-art applications in the clinical setting. Oncologists, pathologists and scientists collaborated to develop innovative ideas about breast cancer research and to enhance its relevance to patient care. This report highlights the most recent discussions in fundamental research and future clinical perspectives presented by professionals from around the world. It also covers the important issues regarding new technologies for biomarker discovery and the actual path to clinical utility.

In recent years, there has been rapid progress in fundamental research followed by an increasing development of new strategies for clinical applications [1]. The IMPAKT 2015 conference, chaired by Nicholas Turner (Institute of Cancer Research, London, UK) and Carsten Denkert (Charité Universitätsmedizin Berlin, Berlin, Germany), focused on new strategies to further accelerate the progress of translational research. The multidisciplinary group of experts debated the current issues in breast cancer research and the future of precision medicine, by setting treatment options into perspective. The overall objective is to understand the disease in order to provide better health outcomes and improve patient care.

## Molecular profiling: the significance of genomic patterns

The conference introduced a series of talks that looked beyond the ‘well-known’ gene expression profiling. Using high-throughput sequencing analysis, Serena Nik-Zainal (Wellcome Trust Sanger Institute, Cambridge, UK) exposed genome-wide mutational signatures and hypermutated regions in human breast cancers [2]. The genomic content and the diverse mutational processes have shown promising inferences for understanding breast cancer history, from the complex etiology to disease evolution [3]. In this direction, the investigation pointed to the existence of several subclonal expansions that represent further steps in cancer development [4]. The extent of subclonal diversification varied among breast tumors and followed spatial patterns. These findings highlight the importance of including the analyses of tumor evolution and subclonal structures in future clinical settings.

One of the recent topics of the conference was the discussion of which mutations are clinically relevant, how to identify them and what the therapeutic implications are. The systematic analysis of mutation frequencies and sequence contexts using whole-exome mutation data from The Cancer Genome Atlas (TCGA) revealed the importance of the APOBEC family of cytidine deaminases as a chronic source of DNA damage in breast tumors. Increased mutation frequencies induced by APOBECs, with emphasis on APOBEC3B, was also highlighted by Reuben Harris (University of Minnesota, Minneapolis, MN, USA) [5,6]. Interpreting the findings, APOBECs are responsible for a large proportion of both dispersed and clustered mutations and may explain how some tumors evolve rapidly and manifest heterogeneity.

On the basis of these studies, thousands of somatic mutations identified across many samples have uncovered molecular signatures underlying the breast cancer history. Therefore, additional investigation of the APOBEC-mediated mutagenesis is required to comprehend major regulations and dependent mechanisms affecting the cell phenotype. This knowledge will further delineate the disease landscape with regard to the patient's clinical outcome. The main challenge, however, is to situate the mutation readouts in the context of anticancer therapies. In the view of future medicine, large-scale molecular profiling will be applied to clinical testing for diagnosis, prognosis and therapy in a complementary and more precise and individualized way than current methods.

## A proteomics approach for dissecting breast cancer

Integrated biology approaches involving both the genome and proteome are essential for addressing system-wide biological questions [7]. Compared with the genome, however, the proteome profile is more complex and highly divergent in time and space. Proteins are the ultimate players that determine the cell phenotype and currently hold a translational promise in science. Using high-throughput techniques, such as protein microarray and mass spectrometry, researchers are capable of analyzing thousands of proteins simultaneously. These are valuable methodologies for the identification of biomarkers with prognostic or diagnostic value, and to inform therapeutic responses.

The new proteomics technologies are in high demand and will remain so for at least the next decade, generating data that are complementary and, in some aspects, unique compared with other ‘omics’ approaches [8]. Additionally, integrating ‘omics’ sources and bioinformatics it will allow the assessment of complex protein networks and intricate signaling pathways. Bringing forward novel strategies, Matthew J Ellis (Baylor College of Medicine, Houston, TX, USA) provided an overview of proteomics analysis for dissecting the breast cancer pathological state. He described the emerging ‘next-generation proteomics’ – with new insights on the global proteome and phosphoproteome – that will enhance the translation of basic discoveries into clinical practice.

## Circulating tumor cell liquid biopsy

Circulating tumor cells have potential as a ‘liquid biopsy’ to evaluate cancer in both the metastatic and early stage setting. There is a need for novel biomarkers with greater sensitivity and specificity to define disease prognosis, and to delineate the benefit of new and emerging therapies as well as treatment response. Bram De Laere (University of Antwerp, Antwerp, Belgium) conducted research to determine the PIK3CA genotype in circulating tumor cells, at the single cell level. In the noninvasive evaluation of blood, PIK3CA was frequently mutated across patients with metastatic hormone receptor-positive breast cancer. The analysis revealed the PIK3CA mutations in both homo- and hetero-geneity in circulating tumor cells, which suggested the presence of subclones with both mutant and wild-type variant in the population.

The PIK3CA status assessed in circulating tumor cells and cfDNA of liquid biopsies paves the way towards novel method for the management of patients with metastatic cancer. A critical question to be answered is how this status changes over time or is influenced by treatment in a larger cohort and real clinical trials [9,10]. The characterization of circulating tumor cells is key to revealing metastatic features and directing chemotherapy, whereas tumor derived cfDNA may also be relevant for early detection of cancers [11]. Further analysis is required to provide accurate information regarding tumor biology and heterogeneity, and to validate the clinical utility of liquid biopsies.

## Biomarkers in drug response & resistance

The explosion of molecular profiling assays has increased the pressure to identify novel markers for clinical implementation. Due to the lower risk of adverse treatment events, some researchers rely on defining more selective inhibitors rather than pan-inhibition agents which affects spread targets. Following this idea, Ian Krop (Dana Farber Cancer Institute, Boston, MA, USA) showed the mechanisms involved in PI3K/AKT/mTOR pathway inhibition and the increased drug sensitivity of ER-positive breast cancer cell lines. The pathway inhibitor also suppresses cyclin D1 expression thereby affecting the CDK4/6, responsible for mediating cell division and proliferation [12]. The combination of PI3K and CDK4/6 inhibition, ultimately, induced apoptosis *in vitro* and *in vivo*.

In tests with palbociclib, an inhibitor of CDK4/6, Maria Teresa Herrera-Abreu (Institute of Cancer Research, London, UK) showed that cell lines acquired resistance following chronic exposure to the drug. The mechanisms underlying resistance may involve the adaptive loss of cyclin D1 dependence or the acquired loss of RB1 phosphorylation. Agents that work synergistically with palbociclib may increase the drugs activity and improve the patient's clinical outcome. Rational drug combinations, however, may increase sensitivity in preclinical tests and further support medical development and cancer care innovation. Large prospective clinical trials to better assess the patients’ outcome is therefore essential in evaluating the drug efficacy and effectiveness.

## Conclusion

The conference provided a stimulating environment for discussing translational research in breast cancer via relevant updates and new insights for understanding the disease. The conference encouraged team integration and enhanced networking opportunities among specialists and early career professionals. The high-level scientific overview and exchange of opinions promoted the latest advances in molecular biology that can be exploited for clinical purposes. Despite the clear impact molecular profiling (genome, transcriptome, proteome) has made in improving our knowledge of breast cancer history, there is still a great deal of work ahead. The findings need to be transformed into clinical applications, either for early diagnosis or effective treatments, in the sphere of personalized medicine.
